# Complex Sleep Apnea Syndrome

**DOI:** 10.1155/2014/798487

**Published:** 2014-02-16

**Authors:** Muhammad Talha Khan, Rose Amy Franco

**Affiliations:** Division of Pulmonary, Critical Care and Sleep Medicine, Medical College of Wisconsin, 9200 W. Wisconsin Avenue, Milwaukee, WI 53226, USA

## Abstract

Complex sleep apnea is the term used to describe a form of sleep disordered breathing in which repeated central apneas (>5/hour) persist or emerge when obstructive events are extinguished with positive airway pressure (PAP) and for which there is not a clear cause for the central apneas such as narcotics or systolic heart failure. The driving forces in the pathophysiology are felt to be ventilator instability associated oscillation in PaCO_2_ arterial partial pressure of Carbon Dioxide, continuous cositive airway pressure (CPAP) related increased CO_2_ carbon dioxide elimination, and activation of airway and pulmonary stretch receptors triggering these central apneas. The prevalence ranges from 0.56% to 18% with no clear predictive characteristics as compared to simple obstructive sleep apnea. Prognosis is similar to obstructive sleep apnea. The central apnea component in most patients on followup using CPAP therap, has resolved. For those with continued central apneas on simple CPAP therapy, other treatment options include bilevel PAP, adaptive servoventilation, permissive flow limitation and/or drugs.

## 1. Introduction

Obstructive sleep apnea syndrome (OSAS) affects a growing proportion of general population affecting both men (15%) and woman (5%) and is commonly known as “sleep disordered breathing” [1]. OSAS is linked with significant cardiovascular morbidity and mortality in those untreated [2]. In OSAS, repetitive collapse of the upper airway takes place, which will finally lead to O_2_ arterial Oxygen desaturation and arousal. Continuous positive airway pressure (CPAP) is the standard therapy to stabilize the airway preventing repeated collapse. To a lesser extent, central sleep apneas syndrome is diagnosed in about 5% of those who undergo a sleep study. This condition is characterized by diminished respiratory regulation during sleep, resulting in decreased or absent ventilation and disturbed gas exchange [3]. Because the mechanism behind the developing the central events is much more complicated, the response to CPAP is often incomplete and may lead to CPAP failure [4, 5]. For some patients who undergo CPAP treatment for OSAS, CPAP therapy leads to the development of recurrent central apneas or even clear periodic breathing. This phenomenon of obstructive events or mixed central and obstructive events with short cycles of obstruction and the incomplete response to positive airway pressure (PAP) due to CPAP treatment related central events has been labeled “complex sleep apnea syndrome” (CompSAS) [6–9].

## 2. Definition

**Figure 1:**
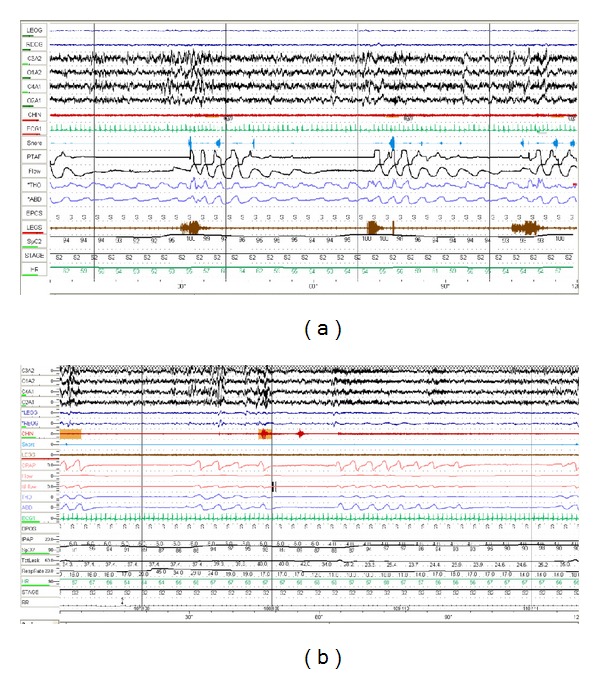
(a) Diagnostic polysomnogram showing obstructive events (arrows). Leads from top to bottom; eye, EEG, chin, ECG, snore, nasal pressure, thermal airflow, thoracic and abdominal effort, legs movement, saturation, and heart rate. (b) Development of central events on CPAP therapy (arrows). Leads from top to bottom; EEG, eye, chin, ECG, snore, legs movement, CPAP, flow, thoracic and abdominal effort, IPAP, saturation, leak, respiratory rate, and heart rate.

Complex sleep apnea syndrome (CompSAS) is a form of sleep disordered breathing in which central apneas persist or emerge when obstructive events have disappeared with PAP therapy (see Figure 1). By the currently accepted definition, the central events must comprise more than half of the residual sleep disordered breathing events or lead to a periodic breathing pattern which on positive airway pressure therapy becomes predominant and disruptive and the central apnea index (CAI) must be > 5 events/hour. Bilevel positive airway pressure ventilation in spontaneous mode may increase the risk of central apneas through augmenting ventilation with a proportionate decrease in carbon dioxide. It is actively being debated whether to include those with central apneas associated with narcotics or Cheyne-Stokes breathing due to systolic heart failure. In the current definition, CompSAS would include only patients whose central apnea could not be diagnosed elsewhere in the central apnea disorders spectrum. In an effort to better define this population and the pathophysiology driving it, these groups are not included in this discussion. While the diagnosis of CompSAS using any criteria will continue to be controversial, there is a need for a diagnostic category for patients with treatment emergent central apneas—especially for those in whom the central apnea does not resolve with chronic CPAP treatment. Complex sleep apnea has also been variably termed CPAP-related periodic breathing [10], complex disturbed breathing during sleep [11], and CPAP-related CSA [12], CSA during CPAP [13].

The prevalence of complex sleep apnea has only recently been evaluated. In a clinical review which did not exclude those with heart failure or narcotic use, 15% of patients (34/223) consecutively evaluated over 1 month for suspected sleep-disordered breathing had CompSAS [9]. However, the prevalence of this syndrome is somewhat variable in part due to the heterogeneous populations being reported/studied and is significantly affected by factors such as narcotic use, BMI, and other comorbidities specially heart failure. The described sleep center prevalence ranges from 0.56% in the Westhoff study from Germany (excluded patients with BNP l > 100 pg/mL) [14] to 18% reported in series of patients with chronic heart failure and OSAS [15].

## 3. Pathophysiology

**Figure 2:**
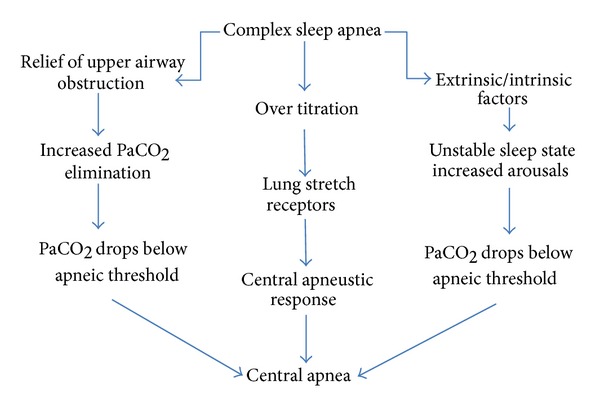
Schematic representation of pathogenesis of complex sleep apnea syndrome.

Differentiating CompSAS from both OSA and CSA arose from the need to characterize the pathophysiology of this syndrome and formulate appropriate management strategies. The pathogenesis is not completely understood but there is thought to be interplay of several factors influencing the pathophysiology (see Figure 2). The key factors are thought to include the interaction of upper airway obstruction and unstable central ventilatory control factors as well as host conditions/characteristics [16, 17]. It has been proposed that CompSAS occurs predominantly during unstable sleep states. In this theory, the development of CompSAS depends on the combination of breathing instability, changes in upper airway related airflow resistance, variable PaCO_2_ levels, and oscillating sleep state. During wakefulness, breathing is controlled by combined behavioral and metabolic factors in addition to central and peripheral chemoreceptors (carotid bodies and medulla). During sleep, the withdrawal of behavioral control of ventilation and blunted chemoresponsiveness to changes in arterial CO_2_ (PaCO_2_) and oxygen (PaO_2_), as well as changes in lung volumes and minute ventilation due to sleep state (REM versus Non REM), leads to more variability in PaCO_2_ levels. In the setting of the recurring upper airway obstructions with OSAS, the variability of PaCO_2_ is even more marked. When chronic recurrent obstruction is resolved by CPAP, ventilation stabilizes improving gas exchange and smoothens the CO_2_ elimination. If the ventilatory response to stimuli is exaggerated, the PaCO_2_ can easily drop below the apneic threshold resulting in a central apnea [18–21]. The central event in turn may trigger an awakening that leads to a sleep/wake state dependent change in ventilator drive and the PaCO_2_ set points for ventilation. Under the right circumstances, this leads to a cyclic phenomenon of central apnea, sleep disturbance, sleep onset, central apnea, sleep disturbance, and so forth. Some patients may have a much tighter apnea threshold to hyperpnoea response (lower breakpoint) making them more susceptible to periodic breathing. It should also be noted that those more likely to demonstrate treatment emergent centrals are also likely to demonstrate central events on polysomnogram during diagnostic portion in addition to predominant obstructive events. Those central events become more prominent during CPAP therapy. Another potential mechanism that would explain central apneas developing with CPAP initiation is the effect of PAP therapy on activation of lung stretch receptors leading to ventilatory changes. When lung stretch receptors are stimulated, a central apnea ensues via the Hering-Breuer reflex [22, 23]. Montesi et al. recently identified nasal masks with inherent leak and mouth leak as potential sources for hypocapnia triggered central apneas in the setting of PAP therapy [24]. Finally, external and internal triggers related to the patients comorbidities and the intrusiveness of CPAP therapy can also trigger frequent arousals, which in turn lead to unstable sleep and oscillation of PaCO_2_. A schematic representation of the pathophysiology is illustrated in Figure 2.

## 4. Clinical Characteristics

Clinically speaking, the phenotype of patients with CompSAS is most similar to that of a patient with OSA, and only when CPAP therapy is applied one can identify them (see Figure 1). From those studies we have in selected populations, it appears that those with complex sleep apnea are more often male (>80% versus 60% with OSA). While it is not clear what the exact mechanism is, increased hypercapnic ventilatory response in males could be playing a role [25]. There is slight higher incidence of ischemic heart disease or heart failure compared to those with OSA [26] but this may be due to bias inherent to the populations being evaluated in these studies. The body mass index (BMI) is typically higher than the general population which is typical in OSAS; however, in one study, the BMI with CompSAS was slightly lower than with OSAS [9]. There may be clinically relevant symptoms that leads to detection of CompSAS. In one study, there was a significant difference in CPAP use and dyspnea compared to those with OSA. The treated CompSAS patients reported more CPAP removal during sleep and dyspnea or air hunger at followup [27]. In fact predicting the presence of CompSAS may not be entirely complete after the initial sleep study. One study reported that, if subject had evidence of central events on baseline study (central apnea index >5), they were more likely to have CompSAS but this was not statistically significant and was not proven by multiple subsequent studies. The severity of sleep apnea present on initial evaluation seems to predict persistent central apneas while on CPAP therapy. Those with severe OSA or CSA on diagnostic study were more likely to have persistent central apneas on subsequent titration studies completed in 2-3 months into ongoing CPAP therapy [28]. The degree of sleep architecture disruption and the hypopnea density in NonREM versus REM sleep may also be predictive. As compare to those with simple OSAS, on subsequent CPAP titration study, those with complex sleep apnea had a higher events/hour index in nonREM sleep compared to REM sleep and had a more elevated arousal index [9].

## 5. Treatment

### 5.1. Positive Pressure Devices

An outline of treatment options is illustrated in Figure 4. Patients with CompSAS can represent a treatment challenge, and the optimal therapeutic approaches remain to be refined. Based on the proposed mechanisms, it appears that the most critical component of any therapy for CompSAS is to minimize potential for developing over ventilation or hypocapnia. This includes targeting CPAP at the lowest pressure that resolves most obstructive events and avoiding modalities that destabilize the airway or sleep state. In a recent retrospective review of 1286 patients receiving PAP therapy, only 6.5% of patients demonstrated CompSAS during the initial CPAP titration study. Of those initially identified with CompSAS, half (*n* = 42) underwent a titration sleep study after an interval of CPAP therapy, and 78.6% (33/42) of those showed resolution of the central apnea component with CPAP [28]. The CompSAS may not be identified on initial sleep studies. The central apneas developed *de novo* in 4% of OSAS patients during followup for CPAP therapy in one retrospective study [29].

Up to this point, CompSAS has been defined by evaluation of an initial sleep study or on followup titration sleep studies. Diagnosing CompSAS will become more challenging as practice protocols are driven out of the controlled environment of the observed sleep study to limited home-based sleep studies followed by autotitrating PAP therapy by the implementation of cost containment in sleep medicine. It is understood that autotitrating PAP devices vary in ability to warn clinician of emerging central apnea. Clinicians will need to be aware of this condition as a possibility in those with either poor response to auto-titrating PAP therapy (lack of improvement in sleep related complaints) or inability to tolerate this type of therapy and champion the need for observed titration testing needed to confirm the presence of CompSAS.

**Figure 3:**
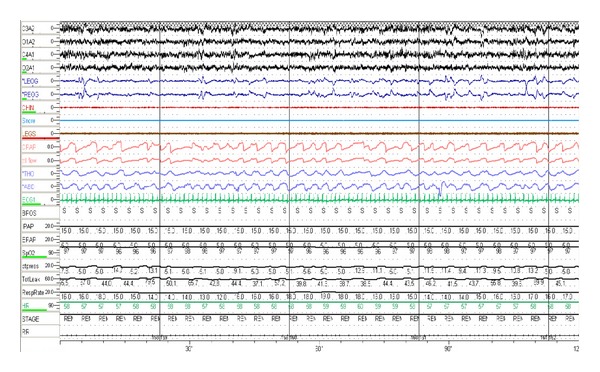
Patient on adaptive servoventilation. Resolution of central events which were noted on CPAP therapy.

**Figure 4:**
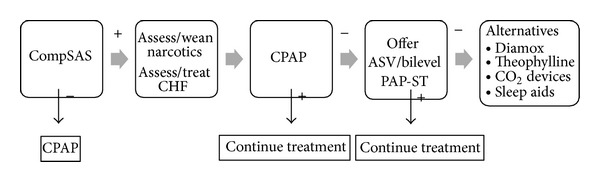
A schematic representation of treatment modalities of complex sleep apnea.

For those with CompSAS who fail CPAP, breaking the cycle of central apneas leading to arousals and instability in ventilation can be achieved with respiratory assist devices designed to better control ventilation. These devices include bilevel PAP in the spontaneous-timed (bilevel PAP-ST) mode and adaptive servoventilation (ASV) [11, 26]. Bilevel PAP-ST is a type of noninvasive ventilation that provides the pneumatic splint effect in the upper airways resolving the obstructive component as well as stabilizing ventilation during central apneas by forcing breaths (timed breath) during episodes of central apneas. The expiratory positive airway pressure (EPAP) is set to eliminate obstructive apneas, while the inspiratory positive airway pressure (IPAP) and back-up ventilation frequency (ST mode) are set in order to decrease hypoventilation [30]. Even more refined ventilator support can be found in ASV devices. This type of bilevel ventilator has been shown to be effective in resolving not only CompSAS but also CSA as well as Cheyne-Stokes respiration and can be measurably effective both in the first night of therapy and over long-term management (Figure 3) [26].

Adaptive servoventilators (ASV) vary by manufacturer on how it accomplishes stabilized ventilation. The most commonly used devices available are of one of two types. The first uses an automatic, minute ventilation-targeted algorithm (VPAP Adapt SV ResMed; Poway, CA, USA) and the second uses an automatic adjustable tidal volume target (BiPAP autoSV; Respironics; Murrysville, PA, USA) which performs a a breath-to-breath analysis and alters settings accordingly [31]. In a recent retrospective study comparing these two servoventilators, there was no significant difference in improvement in AHI [32]. With ASV, the end expiratory pressure is determined during PSG by evaluating the pressure needed to eliminate obstructive apneas. The ASV device automatically determines the extent of the ventilatory support during inspiration (within a set pressure range) based on continuous analysis of the breathing pattern during inspiration [30]. Recent further enhancements on the ASV type of devices include an automatic expiratory pressure adjustment, an advanced algorithm for distinguishing open versus obstructed airway apnea, a modified auto backup rate which is proportional to subject's baseline breathing rate, and a variable inspiratory support.

With these devices ability to assist ventilation with a more natural or gentle push in pressure support that leads to augmentation of the intrinsic ventilation of the patient, there is thought to be less tendency to cause excess ventilation as has been reported to occur with bilevel PAP-ST.

When comparing bilevel PAP-ST with ASV, a prospective randomized crossover clinical trial with patients with CSA/CSR, predominantly mixed apneas and CompSAS in an acute setting, apnea hypopnea index (AHI) and respiratory arousal index (RAI) were markedly reduced with both devices. The AHI and RAI were both statistically lower using ASV as compared to bilevel PAP-ST ((*P* < 0.01) AHI 6.2 versus 6.4, RAI 0.8 versus 2.4) [26]. Clinically significant improvements in ventilatory parameters with the adaptive servoventilators have been measured in other trials. In one recent randomized trial, during initial titration bilevel PAP-ST and servoventilation both significantly improved the AHI, central apnea index (CAI), and oxygen desaturation index (ODI), when compared to CPAP treatment (all *P* < 0.05). However, at six weeks, servoventilation treated respiratory events more effectively than Bilevel PAP-ST in patients with complex sleep apnea syndrome (Bilevel PAP-ST versus servoventilation, respectively: AHI (16.5 ± 8 versus 7.4 ± 4.2 events/h, *P* = 0.027), CAI (10.2 ± 5.1 versus 1.5 ± 1.7 events/h, *P* < 0.0001), and ODI (21.1 ± 9.2 versus 4.8 ± 3.4 events/h, *P* < 0.0001)) [33].

When none of the previously discussed PAP therapies or respiratory assist devices are well tolerated, using lower pressures by allowing some obstruction to persist (permissive flow limitation) may be successful in some patients [11].

### 5.2. Other Treatment Options

The evidence for drug therapy to treat central apneas in CompSAS is limited and includes using drugs that either improve periodic breathing itself (e.g., theophylline or acetazolamide) or increase the percentage of NREM sleep that exhibits stable characteristics (slow wave sleep) [11, 31].

### 5.3. Experimental Therapies

Pulling from the findings that increasing the inhaled percentage of CO_2_ can resolve central apneas (cite), experimentally successful treatment of CompSAS with PAP therapy plus controlling inhalation gases blended with 0.5–1% CO_2_ resulted in an immediate (1 min) decrease in AHI to <5 events/h, without complaints of dyspnea, palpitations, or headache [34]. Safety concerns of gas application have to be addressed before the application of CO_2_ can be considered for clinical use. Finally, increasing the overall dead space or enhancing expiratory rebreathing space in positive airway pressure therapy has been shown to be effective as well in the treatment of CompSAS; again the reports are of a limited number of subjects and for limited duration, raising concerns of generalizablity and sustainablity of this therapy [35, 36].

## 6. Conclusions

Complex sleep apnea syndrome is the diagnostic term for the form of central sleep apnea that persists or develops upon treatment of primarily obstructive sleep apnea with CPAP. The pathogenesis likely is related to a combination of the impact of CPAP therapy on ventilation, disturbed ventilatory control related to sleep and host response, and other medical comorbidities. It is more common in men, coronary artery disease, and those with congestive heart failure. The vast majority will be successfully treated with CPAP but caution is recommended to ensure that the CPAP pressure setting is limited to treating only the obstructive breaths (limited over titration). For those who are poor candidates for CPAP therapy and those who are CPAP therapy failures, more advanced respiratory assist devices including bilevel PAP-ST or adaptive servoventilation therapy can be effective. Other therapeutic medications such as acetazolamide or theophylline may offer an alternative when positive pressure devices of any type are ineffective or poorly tolerated. New devices aimed at increasing the amount of inhaled carbon dioxide gas to stabilize the breathing pattern appear promising and are under development.
